# Imaging Thermoelectric Properties at the Nanoscale

**DOI:** 10.3390/nano11051199

**Published:** 2021-05-01

**Authors:** Stéphane Grauby, Aymen Ben Amor, Géraldine Hallais, Laetitia Vincent, Stefan Dilhaire

**Affiliations:** 1 CNRS, Laboratoire Ondes et Matière d’Aquitaine LOMA, Université de Bordeaux, UMR 5798, 33400 Talence, France; aymen.ben-amor@u-bordeaux.fr (A.B.A.); stefan.dilhaire@u-bordeaux.fr (S.D.); 2 CNRS, Centre de Nanosciences et de Nanotechnologies-C2N, Université Paris Saclay, 91120 Palaiseau, France; geraldine.hallais@c2n.upsaclay.fr (G.H.); laetitia.vincent@c2n.upsaclay.fr (L.V.)

**Keywords:** nanowire, Seebeck, thermal conductivity, electrical conductivity, imaging

## Abstract

Based on our previous experimental AFM set-up specially designed for thermal conductivity measurements at the nanoscale, we have developed and validated a prototype which offers two major advantages. On the one hand, we can simultaneously detect various voltages, providing, at the same time, both thermal and electrical properties (thermal conductivity, electrical conductivity and Seebeck coefficient). On the other hand, the AFM approach enables sufficient spatial resolution to produce images of nanostructures such as nanowires (NWs). After a software and hardware validation, we show the consistency of the signals measured on a gold layer on a silicon substrate. Finally, we demonstrate that the imaging of Ge NWs can be achieved with the possibility to extract physical properties such as electrical conductivity and Seebeck coefficient, paving the way to a quantitative estimation of the figure of merit of nanostructures.

## 1. Introduction

In recent decades, promising strategies have been developed to control the transport of energy at the nanoscale through nanostructuration and the development of nanotechnologies. This involves a wide range of applications, among which are nanoelectronics [1,2], photonics [3,4] and plasmonics [5] or renewable energy systems such a photovoltaics [6,7] or thermoelectricity [8,9].

Indeed, concerning thermoelectricity, nanostructuration can enable the shaping of new materials behaving as “phonon glass and electron crystal”, i.e., a good thermal insulator and good electrical conductor, most specifically in the case of semiconductors. For such materials, at room temperature, phonon mean free paths are usually of the order of a few hundred nanometers, while electron mean free paths are only a few nanometers. Hence, reducing sizes to a few tens or hundreds of nanometers can induce a reduction of the phonon mean free path without impacting the electron mean free path. The thermal conductivity λ is then expected to be reduced [10,11] without impacting the electrical properties (Seebeck coefficient S and electrical conductivity σ) [12].

Nanostructuration processes are becoming more and more advanced (nanowire, nanolayers, nanodots, etc.) [13,14], and, in particular, nanowire arrays might offer significant opportunities for thermoelectricity applications [15,16]. However, there is a lack of methods able to measure the physical properties, particularly the electrical and thermal ones, at the nanoscale. The existing methods can be separated into two categories: on the one hand, methods measuring single isolated nanowires (NWs) and, on the other hand, methods measuring NWs embedded in a matrix which constitutes the actual thermoelectric device. Concerning single isolated NWs, microchip suspended structures or platforms are powerful techniques through their capacity to measure several properties of a wide variety of NWs over a wide temperature range [17,18,19,20]. However, these methods are difficult to implement and the necessity to isolate the individual nanowire, the estimation of the contact resistances and the possible oxidation of the nanowire are other major drawbacks. Scanning probe microscopy (SPM) [21] or optical methods, such as Raman [22] or photothermal techniques [23], are other methods adapted for single isolated NWs measurements. In addition, these two last kinds of techniques are also suited for measuring electrical and thermal properties of NWs embedded in their matrix but, while the spatial resolution of optical methods is limited to 500 nm to 1 µm because of diffraction, SPM techniques can image structures with sizes as small as a few tens or hundreds of nanometers. A detailed review of the different techniques is presented in [24].

Among SPM techniques, scanning thermal microscopy (SThM) [25,26,27] has been widely used to study heat transport in nanostructures and, in particular, by using an active regime called 3*ω*-SThM, one can identify the thermal conductivity of NWs. We have previously developed a SThM set-up which offers the possibility to simultaneously image the thermal conductivity of several individual nanowires embedded in their matrix [28]. We have then studied the thermal conductivity of organic and inorganic NWs [29,30] and, more specifically, we have shown a reduction of the thermal conductivity which can be attributed to the reduction of the NW diameter [31] or to the size of polytype nanodomains in the case of Si 3C/2H heterostructured NWs [32].

However, for a complete thermoelectric characterization, the Seebeck coefficient and electrical conductivity are necessary. Additionally, to date, there has been no experimental benchmark able to simultaneously provide images of both properties of individual NWs embedded in a matrix at the nanoscale. However, Xu et al. [33] have proposed an experimental set-up and demonstrated its ability to measure the Seebeck coefficient of nanolayers.

In the present paper, we hence propose an original AFM bench, which is the first one, to our knowledge, enabling one to simultaneously obtain thermal and electrical property images of several individual nanowires embedded in their matrix with a nanometer spatial resolution. In Section 2, we describe the experimental set-up as well as the analytical expressions of the various electrical signals that we can measure. A validation procedure of these electrical signals is then presented in three steps. First, we propose an electrical simulation procedure (presented in the Supplementary Materials) thanks to both simulation software and an electrical hardware circuit figuring the tip–sample system. Then, in Section 3, in the first part, the validation consists in non-scanning point experimental measurements on a sample made of a gold thin layer deposited on a silicon substrate. Finally, in the second part of Section 3, we use the AFM in its classical scanning regime and present our first experimental images of an NW array sample, demonstrating the ability to obtain electric and thermoelectric images at the nanoscale.

## 2. Materials and Methods

The set-up is based on the principle used for 3*ω*-SThM [28] since we work in a sinusoidal regime and the electronic instrumentation is composed of a Wheatstone bridge, amplification and a lock-in amplifier (SR830 Stanford Research Systems, Sunnyvale, CA, USA). Nevertheless, the configuration has been totally revised to implement additional functionalities.

**Figure 1:**
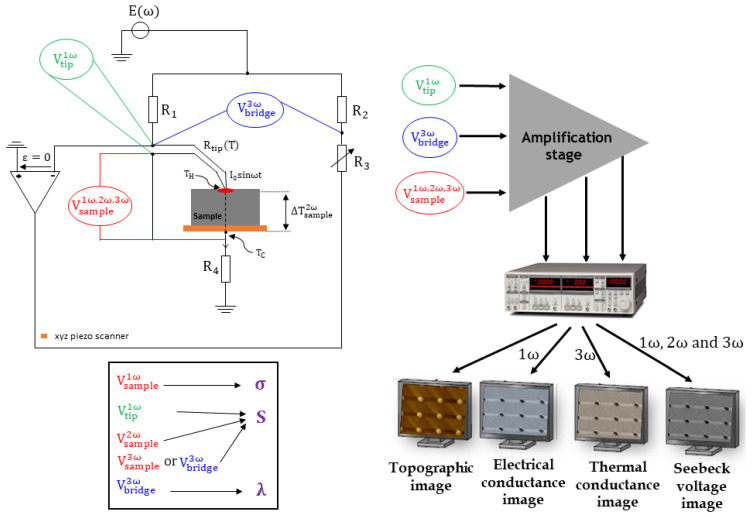
Schematic experimental set-up. Three different voltages are detected and amplified and their spectral components are finally measured by 3 lock-in amplifiers to be displayed by the AFM imaging system. Four images can then be acquired: a topographic image, a thermal conductance image, an electrical conductance image and a Seebeck voltage image.

The schematic experimental set-up is presented in Figure 1. We use a thermoresistive tip in an active regime: it acts like a heat source since it is electrically heated by the Joule effect through the *E*(*ω*) voltage supplying it, which is modulated at pulsation *ω*. Similarly to the 3*ω*-SThM situation, the tip temperature then oscillates at a 2*ω* pulsation with a DC component. We tried two kinds of thermoresistive probes: we first began with a Pd tip classically used for thermal conductivity measurements and, the second time, a Wollaston tip, which is stiffer, to ensure a satisfying contact for electrical measurements, as we will explain later.

The electronic instrumentation has been designed to be able to measure 3 different signals: the tip voltage *V_tip_*, the bridge voltage *V_bridge_* and the sample voltage *V_sample_*. With the Wheatstone bridge used in this configuration, from *V_bridge_*, we can measure the 3*ω* component whose amplitude, when the bridge is equilibrated, i.e., *R*_1_ ≈ *R*_2_ and *R_c_*_0_ ≈ *R*_3_ with *R_c_*_0_ the tip resistance at ambient temperature, is:

(1) $$V_{bridge}^{3\omega }=\frac{R_{2}}{R_{2}+R_{3}}\frac{\alpha R_{c0}\Delta T_{Tip}^{2\omega }}{2R_{1}}E_{0}$$

where *α* is the temperature coefficient of the tip (in K^−1^), $\Delta T_{Tip}^{2\omega }$ the tip temperature variation and *E*_0_ the amplitude of the supplying voltage. For a Wollaston probe, *α* and *R_c_*_0_ are typically around 1.6 × 10^−3^ K^−1^ and 3 Ω, respectively. For a Pd tip, *α* and *R_c_*_0_ are typically around 1.2 × 10^−3^ K^−1^ and 300 Ω, respectively. From this 3*ω* voltage, deducing the tip temperature variations $\Delta T_{Tip}^{2\omega }$, we have already shown that we can deduce the thermal conductivity of the sample under interest [28,29,30,31]. Since we also measure the tip voltage, we can write:

(2) $$V_{bridge}^{3\omega }=\frac{R_{2}}{R_{2}+R_{3}}\frac{\alpha \;\Delta T_{Tip}^{2\omega }}{2}V_{tip}^{1\omega }\;\mathrm{with}\;V_{tip}^{1\omega }=\frac{R_{c0}}{R_{1}}E_{0}$$

It has been checked that the signal obtained with this new version is identical to the one previously measured with the set-up described in [28] providing a standard 3*ω*-SThM. However, now, the real contribution is the richness of the sample voltage V_sample_. Indeed, the set-up enables one to simultaneously measure the different components of this signal. Under the condition that *α*$\Delta T_{Tip}^{2\omega }$ << 1, i.e., $\Delta T_{Tip}^{2\omega }$ << 600 K, these components can be written as:

(3) $$V_{sample}^{1\omega }=\frac{R_{4}}{R_{4}+R_{sample}}\frac{R_{c0}}{2R_{1}}E_{0}=\frac{R_{4}}{2(R_{4}+R_{sample})}V_{tip}^{1\omega },$$

(4) $$V_{sample}^{2\omega }=\frac{R_{4}}{R_{4}+R_{sample}}S\times \Delta T_{sample}^{2\omega },$$

where $\Delta T_{sample}^{2\omega }$ is the sample temperature gradient generated at pulsation 2*ω* along the sample, and

(5) $$V_{sample}^{3\omega }=\frac{R_{4}}{R_{4}+R_{sample}}\frac{\alpha R_{c0}\Delta T_{Tip}^{2\omega }}{4R_{1}}E_{0}=\frac{R_{4}}{R_{4}+R_{sample}}\frac{\alpha \;\Delta T_{Tip}^{2\omega }}{4}V_{Tip}^{1\omega }$$

This experimental set-up is then self-sufficient. Indeed, the electrical resistance of the sample can be deduced from Equation (3):

(6) $$R_{sample}=\frac{R_{4}}{2}\frac{R_{c0}}{R_{1}}\frac{E_{0}}{V_{sample}^{1\omega }}-R_{4}=\frac{R_{4}}{2}\frac{V_{tip}^{1\omega }}{V_{sample}^{1\omega }}-R_{4}$$

Additionally, then, using an electrical model of the tip–sample system, the sample’s electrical conductivity can also be evaluated. We can also estimate the temperature variation of the tip $\Delta T_{Tip}^{2\omega }$:

(7) $$\Delta T_{Tip}^{2\omega }=\frac{2}{\alpha }\frac{V_{sample}^{3\omega }}{V_{sample}^{1\omega }}$$

Then, an estimation of the sample thermal conductivity is possible based on the 3*ω*-SThM previously developed [28].

Finally, the effective Seebeck coefficient of the sample can also be deduced from Equation (4). Nevertheless, it must be noted that an estimation of $\Delta T_{sample}^{2\omega }$ is then necessary. In [33], the authors, using the same experimental set-up with a Wollaston probe, assume that $\Delta T_{sample}^{2\omega }=\Delta T_{Tip}^{2\omega }$. In other words, they consider that the tip-sample contact thermal resistance is negligible. They justify this assumption using a thermal resistance model developed by McGee et al. in [34]. It shows that:

(8) $$\Delta T_{sample}^{2\omega }=\frac{1}{\frac{\lambda_{sample}}{\lambda_{tip}}A+1}T_{2\omega }$$

where $\lambda_{sample}$ is the sample thermal conductivity, $\lambda_{tip}$ is the tip thermal conductivity, *A* is a constant related to the geometry and mechanical properties of the tip. In the case of a Wollaston tip, *A* ≈ 1.12 and $\lambda_{tip}=38\;W\cdot m^{-1}\cdot K^{-1}$. For low thermal conductivity samples, it then can be assumed that $\Delta T_{sample}^{2\omega }=\Delta T_{Tip}^{2\omega }$. However, generally, it must be then taken into account that

(9) $$\Delta T_{sample}^{2\omega }=\beta \;\Delta T_{Tip}^{2\omega }\;\mathrm{with}\;\beta =\frac{1}{\frac{\lambda_{sample}}{\lambda_{tip}}A+1}$$

Combining Equations (4), (5) and (9), we can deduce the effective Seebeck coefficient of the sample:

(10) $$S=\frac{\alpha R_{c0}E_{0}}{4\beta R_{1}}\frac{V_{sample}^{2\omega }}{V_{sample}^{3\omega }}=\frac{\alpha }{4\beta }\times V_{tip}^{1\omega }\times \frac{V_{sample}^{2\omega }}{V_{sample}^{3\omega }}$$

The sample signal *V_sample_* then itself contains all the information necessary to extract the three parameters involved in the factor of merit $ZT=S^{2}\sigma T/\lambda$ with T being the absolute temperature in Kelvin. However, we also implemented voltage outputs to measure 2 other signals: *V_bridge_* and *V_tip_*. Indeed, these voltages can be useful to have a second estimation of the temperature variation $\Delta T_{Tip}^{2\omega }$ from Equation (2). In addition, the Seebeck coefficient can then be deduced from $V_{sample}^{2\omega }$ in combination with either $V_{sample}^{3\omega }$ or $V_{bridge}^{3\omega }$ since both quantities are proportional:

(11) $$V_{bridge}^{3\omega }=\frac{2R_{2}}{R_{2}+R_{3}}\frac{R_{4}+R_{sample}}{R_{4}}V_{sample}^{3\omega }$$

All the measured signals with the possibly deduced physical properties are summarized in Figure 1. The measurements of these voltages combined with the use of an AFM should enable one to evaluate the ZT of nanostructured materials, and in particular of nanowires. The instrumentation system also includes an amplification stage and lock-in amplifiers to isolate the different frequency components of the voltages.

Contrary to the Seebeck coefficient measurement which has been already validated [33], the possible estimation of the electrical conductivity on the same AFM experimental set-up, simultaneously with the other ZT parameter estimations, has never been demonstrated before. In addition, no NW electrical images at the nanoscale have been presented in the literature.

Before completing the measurements on materials, we need then to validate this part of the experimental benchmark. To do so, an electrical simulation procedure, including both simulation software and an electrical hardware circuit figuring the tip–sample system, is presented in the Supplementary Materials.

## 3. Results and Discussion

### 3.1. Experimental Validation on a Gold Layer/Si Substrate Sample

The electronic instrumentation card and the experimental procedure for the electrical resistance are validated, and the next step consists in experimentally validating the measurement of the effective Seebeck coefficient and electrical resistance with the actual tip on a reference sample made of a 100 nm gold layer deposited on a 500 µm p-doped Si substrate. In this section, all the measurements are static measurements with the tip not scanning the sample.

In such conducting AFM techniques, there is a challenge to fabricate probes that are sharp and robust enough to ensure a good electrical contact [35]. As mentioned above, previously, for thermal conductivity measurements, we used a Pd thermoresistive probe presenting two main advantages with respect to the Wollaston tip: a smaller contact radius enabling a better spatial resolution, and a higher thermal cut-off frequency enabling a higher sweeping speed and hence a reduced acquisition time. Nevertheless, after various attempts with Pd probes, it seems that although this kind of probe is well suited for thermal measurements, it is not stiff enough and the contact surface is not large enough to ensure a good electrical contact for electrical and Seebeck measurements. As a consequence, all the experimental measurements presented later have been performed using a Wollaston probe which is also a thermoresistive tip classically used for thermal measurements. Its value is typically 3 Ω at room temperature and its temperature coefficient around 1.6 × 10^−3^ K^−1^. One major drawback of this probe is its spatial thermal resolution, estimated to be about 1 µm [27].

**Figure 2:**
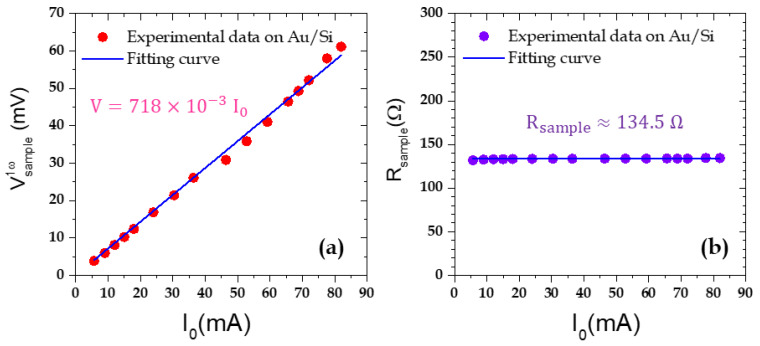
(**a**) Amplitude of the first component of the sample voltage and (**b**) deduced sample equivalent resistance as a function of the supplying current amplitude. The sample voltage behavior is quite linear and the deduced equivalent resistance is very stable across the whole current range, even for current amplitude as low as a few mA.

After checking the consistency of the tip voltage (see Supplementary Materials), we now analyze the other signals from which we can estimate the electrical resistance of the sample and its Seebeck coefficient. Let us first begin with the electrical resistance which has been previously measured by connecting an ohmmeter to the Wollaston output terminals. The value given by the ohmmeter was 135.3 Ω. We then plotted the first harmonic of the sample voltage $V_{sample}^{1\omega }$ as a function of the current amplitude (Figure 2a). Then, from Equation (6), we could deduce the sample resistance value estimated for the different current values. The results are presented in Figure 2b, giving a value *R_sample_* = 134.5 ± 0.2 Ω. The error only corresponds to the uncertainty measured on the regression curve fit. First, we note that, as expected, the identified resistance value is very stable and hence not dependent on the current amplitude. However, another remarkable point is that this value is in very good agreement with the ohmmeter one. Let us underline that this value does not exactly correspond to the sample resistance itself but to a system of several resistances in series: the tip–sample contact resistance, the sample resistance, the sample holder resistance and the resistance of the wire contacting the sample holder. The two last ones are very small and negligible and the resistance of the probe is known. Finally, a tip–sample contact resistance evaluation is then needed to be able to deduce the sample resistance. We will see in the next section that, under certain conditions, we can make a few assumptions to evaluate the electrical resistance of the sample itself but this aim is beyond the scope of this paper and will be the scope of a further work of NW metrology.

The next step consists in the validation of the Seebeck voltage measurement with the aim to possibly later deduce a Seebeck coefficient of the sample. As detailed in the previous section (Equation (10)), the effective Seebeck coefficient can be deduced from the *V_sample_* signal and *V_tip_* signal.

**Figure 3:**
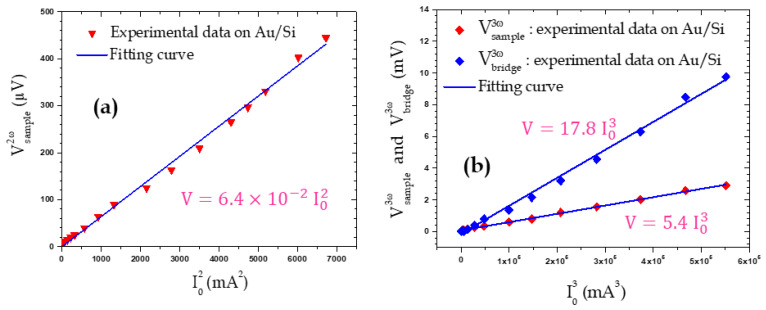
Amplitude of the (**a**) $V_{sample}^{2\omega }$ second and (**b**) $V_{sample}^{3\omega }$ third harmonic of the sample voltage and $V_{bridge}^{3\omega }$ third harmonic of the bridge voltage as a function of the supplying current amplitude. The (**a**) red triangles, (**b**) blue and red diamonds are experimental measurements and the solid blue lines are linear fitting curves.

In Figure 3, $V_{sample}^{2\omega }$, $V_{sample}^{3\omega }$ and $V_{bridge}^{3\omega }$ signals are presented in Figure 3 for different current values. We first note, from the linear behavior, that, as the current increases, $V_{sample}^{2\omega }$ also increases depending on $I_{0}^{2}$. This behavior was expected since, according to Equation (4), this signal depends on $\Delta T_{sample}^{2\omega }$, itself depending on the Joule dissipated power varying as $E_{0}^{2}$ or $I_{0}^{2}$. In contrast, $V_{sample}^{3\omega }$ increases more rapidly depending on $I_{0}^{3}$, as expected from Equation (5). This 3*ω* voltage measurement has been double-checked by also measuring the $V_{bridge}^{3\omega }$ signal. Indeed, from Equations (1) and (5), we can see that both $V_{sample}^{3\omega }$ and $V_{bridge}^{3\omega }$ are very similar. In particular, they are expected to behave similarly relative to E_0_ or I_0_ and they only differ by a multiplying factor (Equation (11)), as shown in Figure 3b. In addition, the 2*ω* tip temperature variations $\Delta T_{Tip}^{2\omega }$ estimated using Equation (7) are also presented in the Supplementary Materials.

**Figure 4:**
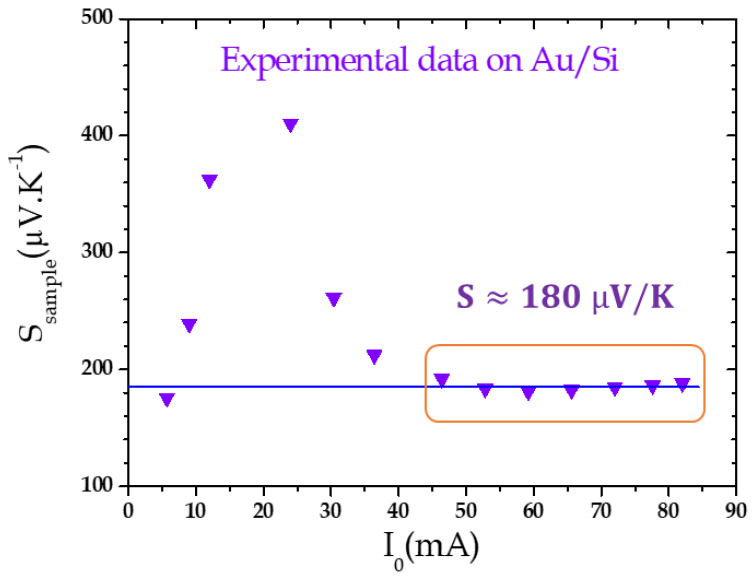
Effective Seebeck coefficient of Au layer on Si substrate versus supplying current amplitude. The identified Seebeck coefficient value becomes stable for current amplitude above 40 mA.

Using Equation (10), it is now possible to deduce the equivalent Seebeck coefficient for different supplying current amplitudes (Figure 4). The *β* coefficient is evaluated to be around 0.1 for the Wollaston tip and the Au thermal conductivity *λ*_Au_ = 317 W m^−1^ K^−1^. For low current amplitudes (below 40 mA), the signal is not stable. This can be explained by the weak signals measured for $V_{sample}^{3\omega }$, and more specifically for $V_{sample}^{2\omega }$, which are only a few tens of µV with a large uncertainty. Then, for currents higher than 40 mA, both $V_{sample}^{3\omega }$ and $V_{sample}^{2\omega }$ become stable and then the effective Seebeck coefficient can be evaluated as 180 ± 2 µV/K. The error only corresponds to the uncertainty measured on the regression curve fit. For this reason, thereafter, the Seebeck measurements are achieved for currents above 40 mA to ensure a sufficient temperature gradient through the sample. Here, again, similarly to the results presented above for the electrical resistance, this coefficient is the global value of the whole system, which includes the Pt/Rh Wollaston half tip, the Au layer, the Si substrate, the sample holder and the connection wires. A model is then needed to reach an accurate determination of the Au layer Seebeck coefficient. However, as a first approximate estimation, we can consider a simple model in which we assume the sample holder, the connection wires and the outer edge of the tip to be at room temperature. Nevertheless, we take into account the Seebeck voltage along the Pt/Rh Wollaston tip with *S_Pt/Rh_* = −4.4 µV/K and a $\Delta T_{Tip}^{2\omega }$ temperature gradient along the tip and the Seebeck sample voltage with a $\Delta T_{sample}^{2\omega }$ temperature gradient along the sample, with both gradients linked through Equation (9). The Seebeck coefficient of the sample can then be deduced from [36]:

(12) $$S_{sample}=\left(V_{sample}^{2\omega }+S_{Pt/Rh}\Delta T_{Tip}^{2\omega }\right)/\Delta T_{Sample}^{2\omega }$$

We can then evaluate the Au/Si sample effective Seebeck coefficient to be around 46 ± 1 µV/K. It is not straightforward to compare this value with the Seebeck coefficient of Au layers which is evaluated to be around 2 µV/K [37] or with the one of Si which, in our case, for the corresponding doping concentration, is evaluated to be around 700 µV/K. A more precise Seebeck model is then needed and is planned to be developed in order to give a more accurate evaluation but also the Seebeck value of the Au layer itself.

Although Seebeck measurements have been previously presented on nanolayers [33,38,39,40], no image of nanostructures, such as NWs, with a nanometer resolution, has been published. The next section will then demonstrate the ability to obtain Seebeck values with the fruitful possibility, as we already did for thermal conductivity measurements, to simultaneously probe several NWs in the same image. In addition, we will also present NW electrical resistance images which can be obtained simultaneously on the same benchmark.

### 3.2. Electrical Measurements of Ge Nanowires

The sample under test is an ordered array of Ge NWs obtained by top-down etching of an n-type (111) Ge substrate, achieved by combining electron beam lithography and reactive ion etching. The patterns consist of a 330 × 120 µm^2^ matrix defined by polygon features of 400 nm in diameter and a 1.6 µm pitch. The deep etch of the patterned substrate was realized by reactive ion etching inductance coupled plasma (RIE-ICP) up to a 1 µm depth. The resulting features are defined by nanopillars with steep edges and a 400 nm diameter. The nanostructures were finally cleaned in hot acetone (50 °C) for a few minutes to remove all residues. After synthesis, Ge NWs were embedded in a hardening hydrogen silsesquioxane resist (HSQ) followed by a baking at 500 °C, transforming HSQ into SiO_2_. For the AFM scanning, the surface of the sample was finally polished using a chemical mechanical polishing (CMP) process with a colloidal silica suspension. SEM images are presented in the Supplementary Materials.

**Figure 5:**
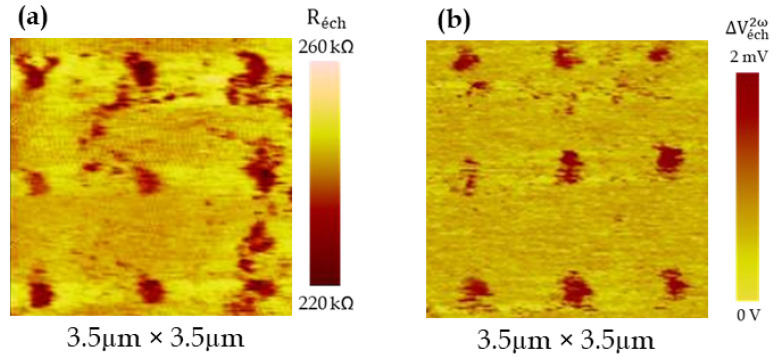
Ge NWs: (**a**) $V_{sample}^{1\omega }$ electrical conductance image and (**b**) $V_{sample}^{2\omega }$ Seebeck voltage image obtained with a Wollaston probe.

The thermal conductivity of such NWs has already been evaluated using the previous system described in [28] and the thermal conductivity images can be found in [32]. We first checked that the thermal signal measured with the new experimental set-up was identical. Then, we scanned the sample with the Wollaston probe and recorded the amplitude of both $V_{sample}^{1\omega }$ and $V_{sample}^{2\omega }$ signal images (Figure 5), which correspond to an equivalent electrical conductance and an effective Seebeck image, respectively. Unexpectedly, the NWs were clearly detected with the Wollaston probe while the spatial resolution was expected to be around 1µm. Nevertheless, this can be explained by the fact that the spatial resolution is either limited by the electrical contact surface for the electrical conductance imaging and by the larger thermal exchange surface for the Seebeck voltage imaging. The thermal exchange radius was measured using the procedure described in [41] and evaluated to be around 750 nm. In addition, the NW pitch was 1.6 µm, larger than the exchange radius, which made the detection of individual NWs possible.

**Figure 6:**
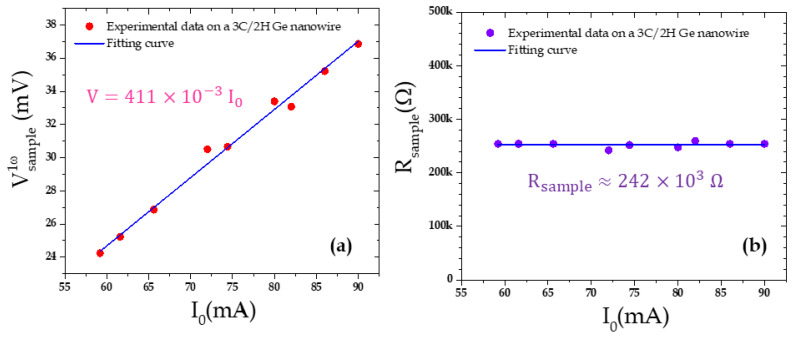
(**a**) $V_{sample}^{1\omega }$ signal measured on a single NW and (**b**) deduced sample resistance as a function of the current. The signals presented correspond to the mean values measured for the 9 NWs visible in Figure 5a.

The contrast is indeed clearly visible both for the electrical conductance and for the Seebeck signals between the matrix and the nanowires. Then, from Figure 5a, stopping the scan of the sample, we positioned the tip on top of one of the nine detected NWs and we measured the $V_{sample}^{1\omega }$ signal when varying the supplying current amplitude (Figure 6a). The behavior was quite linear and we could finally deduce (Equation (6)) the sample equivalent electrical resistance *R_sample_* = 242 ± 4 kΩ (Figure 6b).

A precise modeling of the various electrical resistances of the set-up, in particular of the tip–sample system, is then necessary to evaluate the NW electrical conductivity. Nevertheless, we can roughly estimate it, or at least a range of electrical conductivity. To do so, let us recall that the tip–sample system can be modeled by a contact resistance in series with the NW resistance. We assume that either the contact resistance is negligible or, on the other hand, it is predominant and assimilated into the smallest equivalent resistance measured on the various NWs in the $V_{sample}^{1\omega }$ image. For this second hypothesis, we used an image with 25 NWs. Then, the NW resistance could vary from 82 to 242 kΩ and, taking into account the length and diameter of the NWs, we could deduce that the estimated electrical resistivity of the NWs varied from 2.1 to 6.1 Ω cm. This value is consistent with the resistivity of the bulk N-doped Ge used to process the NWs which were in the range 1–9 Ω cm, corresponding to a low doping concentration (≈10^15^ cm^−3^). The very few results published concerning electrical conductivity measurements on Ge NWs [42,43] are hardly comparable with the ones presented in this paper because of the different doping concentrations or NW diameters. Nevertheless, the fact that we found a value in the range of the furnisher bulk value is consistent since the NWs are too big to induce a possible electrical conductivity reduction, their diameter being far larger than the electron mean free path.

**Figure 7:**
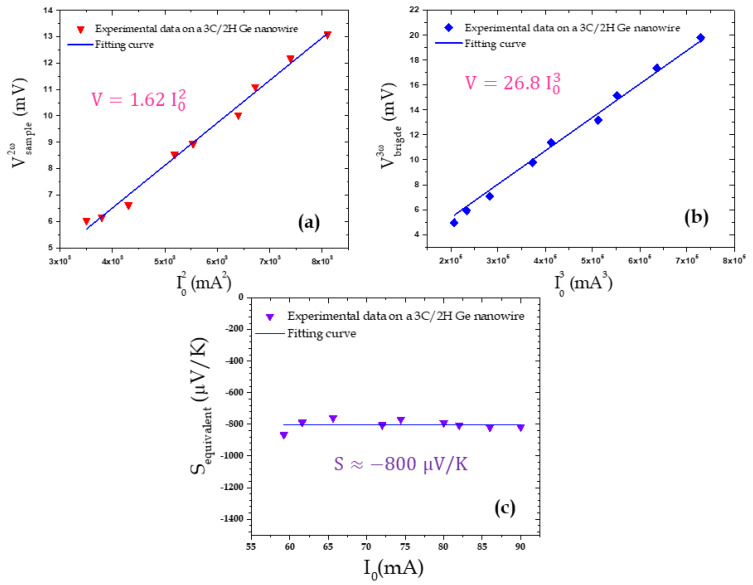
(**a**) $V_{sample}^{2\omega }$ and (**b**) $V_{bridge}^{3\omega }$ signals measured on a single NW and (**c**) deduced equivalent Seebeck coefficient as a function of the current. The signals presented correspond to the mean values measured for the 9 NWs visible in Figure 5b.

Finally, from the $V_{sample}^{2\omega }$ image (Figure 5b), we plotted the $V_{sample}^{2\omega }$ signal measured on an individual NW as well as the $V_{bridge}^{3\omega }$ signal when varying the supplying current amplitude (Figure 7a,b). As expected, the $V_{sample}^{2\omega }$ and $V_{bridge}^{3\omega }$, respectively, depend on $I_{0}^{2}$ and $I_{0}^{3}$. Then, from Equations (10) and (11), we can deduce the effective Seebeck coefficient presented in Figure 7c.

We can then evaluate the Ge NWs’ sample effective Seebeck coefficient to be around −800 ± 10 µV/K. The β coefficient is evaluated to be around 0.67 with a Ge NW thermal conductivity *λ*_Ge_ = 16 W m^−1^ K^−1^ identified in [32]. Using the same simple model as for the Au/Si sample, we can deduce the Seebeck coefficient of Ge NWs/Ge substrate to be −240 ± 3 µV/K. To date, Seebeck coefficients of Ge structures, and in particular Ge NWs, have not abounded in the literature. The Ge Seebeck coefficient has been evaluated at room temperature in the −600 to −1000 µV/K range [44,45,46] for bulk Ge for a low doping concentration and −400 µV/K for Ge thin films [46] but no value could be found for Ge NWs.

## 4. Conclusions

In this paper, we have upgraded and deeply modified our previous SThM set-up to propose an experimental set-up able to image the thermal and electrical properties at the nanoscale from the measurement of the spectral components of three different voltages. The validity of the measured voltages has been checked, first by a software electrical simulation, then by a hardware electrical simulation and finally by experimental measurements on a sample made of an Au/Si stack sample.

Then, it was demonstrated that this set-up could enable us to image individual NWs embedded in a SiO_2_ matrix. In addition to the thermal conductance, we can now image the electrical conductance and Seebeck voltage of nanostructured samples, such as nanolayers or embedded NWs on a substrate, paving the way to a quantitative measurement of the Seebeck coefficient and electrical conductivity of individual NWs.

With that purpose, the next step will consist in devising models of the electrical transport through the samples in order to access the Seebeck coefficient and electrical conductivity of the nanostructure itself, whether it be a nanolayer or a single NW. It should then be possible to evaluate the figure of merit ZT of such nanostructures.

## Data Availability

The data presented in this study are available on request from the corresponding author.

## Supplementary Materials

The following are available online at https://www.mdpi.com/article/10.3390/nano11051199/s1. Figure S1. TINA simulation of the first harmonic component amplitude of (a) the sample voltage versus supplying voltage and (b) tip voltage as a function of the supplying current. From Figure S1a, we can estimate the sample resistance (Equation (6)) and from Figure S1b, we can estimate the tip resistance (from Equation (2)), Figure S2. First harmonic component amplitude of the tip voltage as a function of the supplying current amplitude. The behaviour is quite perfectly linear, Figure S3. $\Delta T_{Tip}^{2\omega }$ tip temperature variations amplitude as a function of the supplying current intensity *I*_0_, Figure S4. SEM images of the Ge NW array sample before (Figure S4a) and after encapsulation in the matrix and polishing (Figure S4b).
