# Supplementary material for: Imaging Thermoelectric Properties at the Nanoscale
Source: Nanomaterials (Basel). 2021 May 1;11(5):1199. doi: 10.3390/nano11051199 (PMC8147288; doi:10.3390/nano11051199)
Supplement: Supplementary file 1 [file nanomaterials-11-01199-s001.zip › nanomaterials-1204794-supplementary-final.pdf]

Supplementary Materials for

Imaging Thermoelectric Properties at the Nanoscale

Stéphane Grauby [1,*], Aymen Ben Amor [1], Géraldine Hallais [2], Laetitia Vincent [2] and Stefan Dilhaire [1]

1 CNRS, Laboratoire Ondes et Matière d'Aquitaine LOMA, Universitéde Bordeaux, UMR 5798,33400 Talence, France; aymen.ben-amor@u-bordeaux.fr (A.B.A.); stefan.dilhaire@u-bordeaux.fr (S.D.)

2 CNRS, Centre de Nanosciences et de Nanotechnologies-C2N, UniversitéParis Saclay, 91120 Palaiseau, France;geraldine.hallais@c2n.upsaclay.fr (G.H.); laetitia.vincent@c2n.upsaclay.fr (L.V.)

* Correspondence: stephane.grauby@u-bordeaux.fr

Validation procedure

We propose an electrical simulation procedure thanks to both a simulation software and an electrical hardware circuit figuring the tip-sample system. It has been first implemented in the TINA (Toolkit for Interactive Network Analysis) software in order to simulate its behaviour. The R_{c0}, R_4 and R_{sample} values have been fixed to 300.0 Ω, 100.0 kΩ and 477kΩ respectively. The 300 Ω thermal tip resistor value has been chosen since it corresponds to the classical value for Pd thermoresistive probes we used to employ for thermal conductivity measurements. Both $V_{tip}^{1\omega}$ and $V_{sample}^{1\omega}$ have been measured and plotted (figure S1) as a function of either the excitation voltage amplitude E_0 or the excitation current amplitude $I_0 = \frac{E_0}{R_1}$. As expected, the behaviour is linear and from the slopes of both curves, we can deduce (see equations(2) and (6)) the identified sample resistance value and the tip resistance value to 477.3 kΩ and 300.0 Ω respectively, in excellent agreement with the input values.

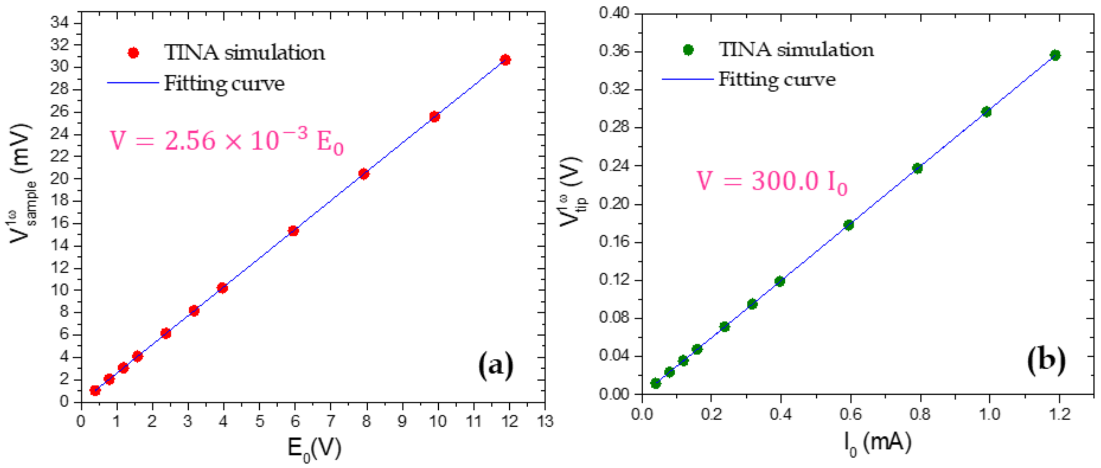

Figure S1. TINA simulation of the first harmonic component amplitude of (**a**) the sample voltage versus supplying voltage and (**b**) tip voltage as a function of the supplying current. From figure(**a**), we can estimate the sample resistance (equation(6)) and from figure(**b**), we can estimate the tip resistance (from equation(2)).

One step further, we have now simulated the tip-sample system by resistor devices and we have used the electronic instrumentation card connected to lock-in amplifiers measuring the $V_{tip}^{1\omega}$ and $V_{sample}^{1\omega}$ amplitudes. The tip and sample have been replaced by 300.4 Ω and 477.2 kΩ resistors respectively and E_0 has been adjusted to 5.67V. The other resistor values are $R_1 = R_2 = 10.1$ kΩ and $R_4 = 99.3$ kΩ. Then, $V_{tip}^{1\omega}$ and $V_{sample}^{1\omega}$ amplitudes are measured to be 170.1 mV and 14.6 mV respectively. We have then

deduced from equations (2) and (6) the values of R_{c0} and R_{sample} to be 303 Ω and 479.1 kΩ respectively. The results are in very good agreement with the expected values with an error lower than 1%.

Tip Voltage Validation on the Au/Si Sample

The Wollaston tip being in contact but not scanning the Au/si sample, we then first analyse the $V_{tip}^{1\omega}$ as a function of the current I_0 amplitude passing through the probe. Then the slope directly gives the tip resistor value. From the results presented in Figure S2, we can deduce the experimental R_{c0} value to be evaluated to 2.84 Ω to be compared with the value measured with an ohmmeter at room temperature which was 2.90 Ω.

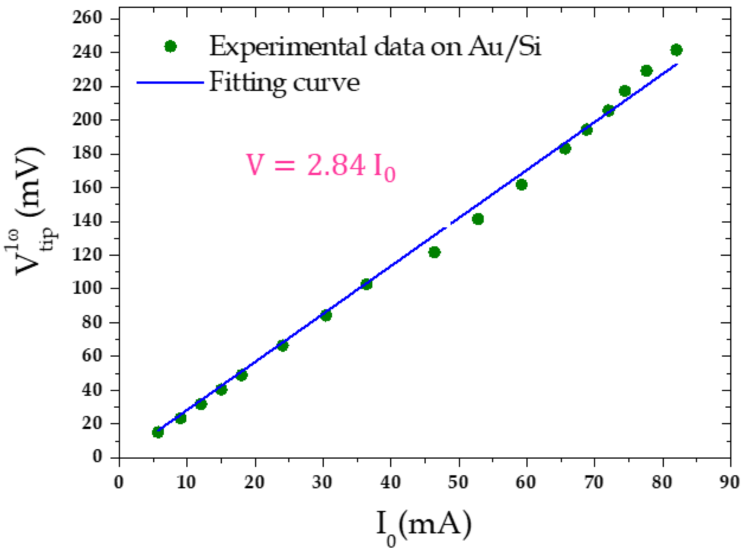

Figure S2. First harmonic component amplitude of the tip voltage as a function of the supplying current amplitude. The behaviour is quite perfectly linear.

Temperature variations signal on the Au/Si sample.

To check the consistency of the various signals we can measure with our experimental system on the Au/Si sample presented in part III, we have evaluated the 2ω tip temperature variations $\Delta T_{Tip}^{2\omega}$ either from $\Delta T_{Tip}^{2\omega} = \frac{2}{\alpha}\frac{V_{sample}^{3\omega}}{V_{sample}^{1\omega}}$ or $\Delta T_{Tip}^{2\omega} = \frac{2}{\alpha}\frac{R_2 + R_3}{R_2}\frac{V_{bridge}^{3\omega}}{V_{tip}^{1\omega}}$ (equations (2) or (7) in the manuscript).

Measuring this quantity as a function of the supplying current, we obtain the curve presented in figure S3. For this kind of probe, the amplitudes of the temperature variations are in good agreement with the ones measured by Lefevre et al in [24].

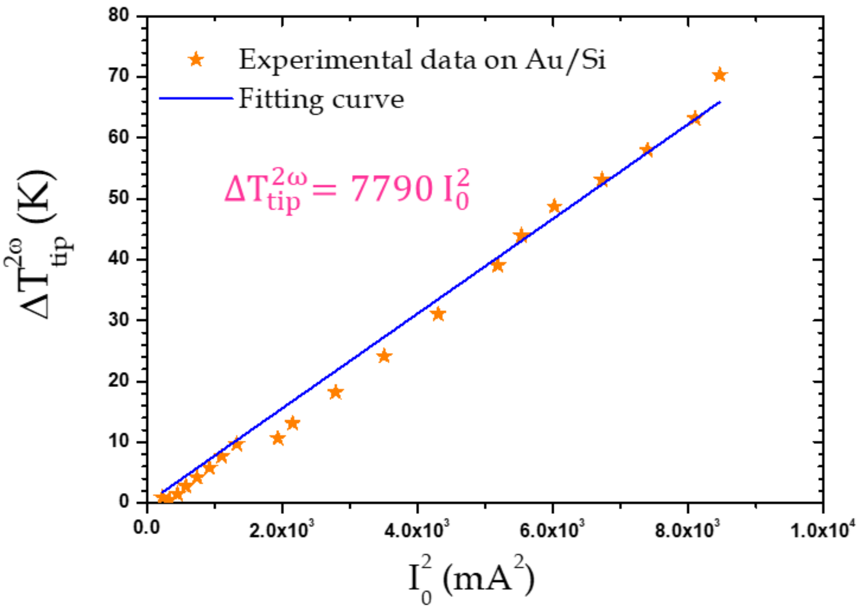

Figure 3. Tip temperature variations amplitude as a function of the supplying current intensity I_0.

SEM Images of the Ge NW Array Sample

The sample under test is an ordered array of Ge NWs obtained by top-down etching of a n-type (111) Ge substrate, achieved by combining electron beam lithography and reactive ion etching. The patterns consist of 330 × 120 μm² matrix defined by polygon features of 400 nm diameter and a 1.6 μm pitch. SEM images are presented in Figure S4.

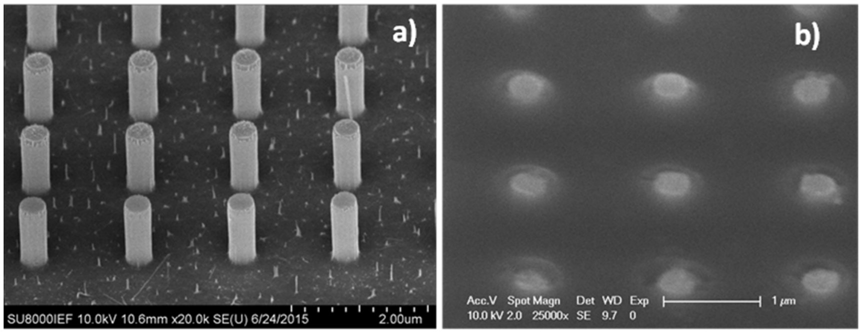

Figure S4. SEM images of the Ge NW array sample before (Figure S4(**a**)) and after encapsulation in the matrix and polishing (Figure S4(**b**)).
